# Effects of polyphenol‐rich traditional herbal teas on obesity and oxidative stress in rats fed a high‐fat–sugar diet

**DOI:** 10.1002/fsn3.2695

**Published:** 2022-01-11

**Authors:** Neelam Iftikhar, Abdullah Ijaz Hussain, Shahzad Ali Shahid Chatha, Nazia Sultana, Hassaan Anwer Rathore

**Affiliations:** ^1^ Department of Chemistry Government College University Faisalabad Faisalabad Pakistan; ^2^ Central Hi‐Tech Lab Government College University Faisalabad Faisalabad Pakistan; ^3^ Department of Pharmaceutical Sciences College of Pharmacy QU Health Qatar University Doha Qatar; ^4^ Biomedical and Pharmaceutical Research Unit (BPRU) QU Health Qatar University Doha Qatar

**Keywords:** BMI, GSH, high‐fat diet, kidney index, LDL and HDL, liver index, MDA, nutraceutical, phenolic acids and flavonoids, SOD

## Abstract

*Hibiscus rosa‐sinensis* and *Zingiber officinalis* teas are traditionally used for the therapies of various diseases, including obesity. The present research work was planned to appraise the potential of polyphenol‐rich extracts of selected herbal plants in obesity and related biochemical parameters of high‐fat–sugar diet‐induced obese rats. Three herbal teas were prepared from *Hibiscus rosa‐sinensis* flowers and *Zingiber officinalis* rhizomes and their mixture (3:1, respectively). Total phenolic contents (TPC) of *Hibiscus rosa‐sinensis* and *Zingiber officinalis* extracts were found to be 5.82 and 1.45 mg/g of dry plant material, measured as GAE, while total flavonoid contents (TFC) were 9.17 and 1.95 mg/g of dry plant material, measured as CE, respectively. Two doses (250 and 500 mg/kg BW) of each tea were administered and body weight, BMI, kidney, liver, and atherogenic indices, TC, TG, HDL, LDL, VLDL, BT, AST, ALT, AP, SC, MDA, SOD, GSH, and TAC of rats groups were measured. Data showed that higher doses of *Hibiscus rosa‐sinensis* significantly reduced the rat's BMI (0.50 g/cm^2^) in comparison with the high‐fat–sugar diet group (0.79 g/cm^2^). All treatment groups, especially H‐500 group, showed a significant decrease in the elevated kidney and liver weights and atherogenic index in comparison with HFSDC groups. Higher doses of *Hibiscus rosa‐sinensis* significantly decreased the levels of AST, ALT, AP, and SC in comparison with the HFSDC group. A significant decrease in the levels of serum TC, TG, LDL, and VLDL was observed in all the treatment groups in comparison with the HFSDC group. Furthermore, all the teas, especially higher doses of *Hibiscus rosa‐sinensis*, prevented the alterations in MDA, SOD, and GSH levels of experimental groups, thus showing the potential against oxidative stress. It can be concluded from these results that *Hibiscus rosa‐sinensis* teas exhibited strong protective effects against obesity and oxidative stress, especially at higher doses.

## Graphical abstract

Results of the present work demonstrated that the use of *Hibiscus rosa‐sinensis* L. and *Zingiber officinale* Roscoe herbal teas at doses of 250 and 500 mg/kg shows anti‐obesity and anti‐oxidative effects in vivo that were more pronounced than a standard anti‐obesity drug (orlistat).

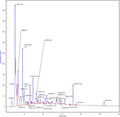

## 1 BACKGROUND

Incessantly increasing rate of unnecessary weight gain and obesity has become a worldwide concern in the past 20 years (NCD‐RisC, 2019). As per the World Health Organization report, about 650 million people in the world are obese and about 1.9 billion adults are overweight (World Health Organization, 2017). Obesity is considered the fifth leading risk factor of mortality as the relative death rate reaches about 2.8 million every year due to the various complications linked with obesity, including oxidative stress, hypercholesterolemia, hypertension, certain cancers, nonalcoholic fatty liver, and coronary heart diseases (Gomathi et al., 2008; Goyal & Kadnur, 2006; Gutin, 2020; Saravanan et al., 2014).

Obesity results from excessive deposition of fats in adipose tissue, pancreatic islets, muscles, liver, and other metabolism‐involved organs. Moreover, the frequency of obesity depends on various factors, including change in lifestyle, genetics, employment and social status, avoidance of physical activities, and increasing consumption of high‐calorie foods (Goyal et al., 2006). Different practices are in use, including various types of surgeries, strenuous exercises, use of herbal formulations, and powerful synthetic drugs such as orlistat, rimonabant, and sibutramine to control obesity‐related issues (Chien et al., 2016). However, there are certain difficulties in the implementation of these therapeutic approaches because of the busy and easy life routines of the individuals. Furthermore, the high cost of the conventional drugs, their toxicities, and unavailability in many rural areas may limit their overall benefits (Chien et al., 2016; Mahmoud & Elnour, 2013). Hence, convenient and effective antiobesity approaches are required to address this issue.

Various medicinal plants and herbs have long been recognized as a chief natural product source and are therapeutically effective against oxidative stress and obesity (Chien et al., 2016; Gomathi et al., 2008; Ismail, 2014; Sobhy et al., 2017; Priya & Veeranjaneyulu, 2016; Sultana et al., 2007). Due to fewer side effects in comparison with synthetic drugs, herbal teas (hot decoction, infusions, and herbal drinks), including lemon, hibiscus, ginger, mint, and cardamom teas, are some of the well‐known beverages. These herbal teas are used not only as refreshment drinks but also as traditional remedies for various ailments, including obesity (Chien et al., 2016; Lingesh et al., 2019; Paranjpe et al., 1990; Priya & Veeranjaneyulu, 2016). Phenolic compounds, due to their antioxidant potential, have shown various biological and pharmacological activities (Hussain et al., 2013; Shah et al., 2020). Therefore, polyphenol‐rich plant extracts have gained much interest by pharmacologists due to various potential biological activities (Shah et al., 2017).

In the present research work, two well‐known traditional teas [*Hibiscus rosa‐sinensis* (Gurhal or Hibiscus tea) and *Zingiber officinale* (Adrak or Ginger tea)] were selected after a thorough ethno‐pharmacological survey to investigate, compare, and validate their potential against obesity (Afify & Hassan, 2016; El‐Rokh et al., 2010; Gomathi et al., 2008; Goyal & Kadnur, 2006; Lingesh et al., 2019; Paranjpe et al., 1990). A herbal decoction of *Hibiscus rosa sinensis* flowers has been traditionally used to reduce body weight (Lingesh et al., 2019) and Shunth, an ayurvedic formulation of *Zingiber officinale*, has been used traditionally for decades in the treatment of obesity (Paranjpe et al., 1990). Some data are available in the literature covering the antiobesity potential of hibiscus and ginger teas (Gomathi et al., 2008; Goyal & Kadnur, 2006). However, to the best of our knowledge, the effects of *Hibiscus rosa‐sinensis* and *Zingiber officinalis* teas in combination and individually at two incremental doses (250 and 500 mg/kg BW) on reduction in body weight gain, BMI, and related biochemical parameters in high‐fat–sugar diet‐induced obese rats are being reported for the first time along with complete phenolic profile. Therefore, the aim of the study was to investigate the phenolic profile and in vivo antiobesity activity of *Hibiscus rosa‐sinensis* and *Zingiber officinale* teas individually and in combination (3:1) using a specific high‐fat–sugar diet‐induced obesity model in WKY rats. Body weight (BW), body mass index (BMI), kidney, liver and atherogenic indices (KI, LI, AI), biochemical (total cholesterol; triglycerides; high‐density lipoprotein; low‐density lipoprotein; very low‐density lipoprotein, bilirubin total; alanine aminotransferase; aspartate aminotransferase; serum creatinine; alkaline phosphatase) and oxidative stress (malondialdehyde; superoxide dismutase; reduced glutathione; total antioxidant capacity) parameters, and histopathological analysis were performed to confirm the effectiveness of selected herbal teas.

## 2 MATERIALS AND METHODS

### 2.1 Collection and identification of plant materials

The petals of *Hibiscus rosa‐sinensis* L. (hibiscus) and rhizomes of *Zingiber officinale* Roscoe (ginger) were collected in the early summer of 2019 from the Herbal Botanical Garden of Government College University, Faisalabad, Pakistan. All plant materials were further identified and authenticated by a taxonomist, by comparison with authentic vouchers (*Hibiscus rosa‐sinensis* L. No 230‐bot‐19 & *Zingiber officinale* Roscoe No. 240‐bot‐19) of botanical herbarium of the university. Authenticated samples were stored in polythene bags and transferred to the Natural Products Research Laboratory, Government College University, Faisalabad, Pakistan.

### 2.2 Reference compounds, reagents, and chemicals

Phenolic and flavonoid standards (caffeic acid, *p‐*coumaric acid, ferulic acid, chlorogenic acid, gallic acid, sinapic acid, *p‐*hydroxy benzoic acid, vanillic acid, catechin, myricetin, quercetin, kaempferol), orlistat, linoleic acid (60%–74%), Folin–Ciocalteu reagent, and ascorbic acid were procured from Sigma Chemical Co. All other chemicals used were of analytical grade and purchased from Merck, unless stated otherwise.

### 2.3 Preparation of herbal teas

The plant materials were cleaned with distilled water and dried in a hot air oven for 10 days (IM‐30m Irmeco). The dried plant samples were ground (mesh size 80) using electric grinder (LG BL 999SP, Germany) and stored at room temperature (25°C). For the preparation of tea, a 28‐g grinded sample was shaken in 280‐ml distilled water (1:10 ratio) separately for each tea and for the preparation of combination tea, 21‐g hibiscus and 7‐g ginger were taken in 280 ml of distilled water. This mixture was selected on the basis of preliminary evaluations. Samples were kept in a temperature‐controlled shaker (Gallenkamp) for 24 h at 50°C temperature for continuous agitation at 180 rpm. Solid residues were separated and the extracts were dried under reduced temperature and pressure using a rotary evaporator to get dried extracts. The yield of extracts was calculated using the formula given below. The material was then saved at −4°C temperature (Hussain et al., 2013).

$$\text{Yield}\left(\frac{g}{100g}\right)=\frac{\text{Weight}\;\text{of}\;\text{dry}\;\text{extract}}{\text{Weight}\;\text{of}\;\text{dry}\;\text{plant}\;\text{material}}\times 100$$

### 2.4 HPLC analysis for phenolic acids and flavonoids

Stock solutions of all the standards were newly prepared by dissolving 1 mg of each reference compound in 1 ml of methanol. Working standards (0.4–100 µg/ml in methanol) were prepared and the calibration curve of each standard was formed. The hydrolysis of herbal tea extracts was done as reported previously (Hussain et al., 2013). The extracts were passed through a nonpyrogenic filter (0.45 µm) prior to injection. The HPLC analysis was performed with Flexar Perkin Elmer System (Perkin Elmer) equipped with gradient model Flexar pumps system, LC‐Shelton CT, 06484 (USA) UV/Visible detector, column oven and degasser (DG‐20A5) systems. A hypersil GOLD C_18_ (250 × 4.6 mm × 5 µm) column (Thermo Fisher Scientific Inc.) and a nonlinear gradient (acetonitrile:methanol (70:30) and water with 0.5% glacial acetic acid) were used. Spectra were recorded at 275 nm and analyses were identified by matching the retention times and spiking the samples with standards, whereas quantification was based on an external standard method.

### 2.5 Determination of antioxidant potential of herbal teas

#### 2.5.1 Determination of total phenolic (TP) and total flavonoid (TF) contents

Total phenolic contents (TPC) of selected herbal teas were measured using Folin–Ciocalteu reagent as reported (Hussain et al., 2013). The standard curve of gallic acid (10–80 ppm) was prepared (*y* = 0.026*x* + 0.000, *R* ^2^ = 0.997) and results were calculated and reported as mg of phenolic content per gram of dry plant weight, measured as gallic acid equivalent.

The total flavonoid contents of selected herbal teas were determined using the method reported by Hussain et al. (2013). The standard curve of catechin (10–160 ppm) was prepared (*y* = 0.006*x* + 0.015, *R* ^2^ = 0.999) and results were calculated and reported as mg of catechin per gram of dry plant weight, measured as catechin equivalent.

#### 2.5.2 DPPH radical scavenging assay

2,2‐Diphenyl‐1‐picrylhydrazyl (DPPH) radical scavenging activity was performed by the method reported by Hussain et al. (2013). The extract and BHT solutions (10 µg/ml) were mixed with an equal volume of 90 μmol/L DPPH solution in methanol. The solution was incubated for half an hour at 30°C, the absorbance was measured at 517 nm, and the percentage scavenging was calculated as follows:

$$\text{Scavenging}\left(\%\right)=\frac{\text{Absorbance}\;\text{of}\;\text{DPPH}\;\text{solution}‐\text{Absorbance}\;\text{of}\;\text{sample}\;\text{solution}}{\text{Absorbance}\;\text{of}\;\text{DPPH}\;\text{solution}}\times 100$$

### 2.6 In vivo antiobesity activity

All animal‐related experiments were performed with prior approval and were carried out in agreement with the procedures of the Institutional Review Board for Animal Studies (Study No. 19680/IRB No 680), Government College University Faisalabad, Pakistan.

#### 2.6.1 Animals

Weaning, 3‐week‐old male Wistar Kyoto (WKY) rats (weighing approximately 130–160 g) were purchased from animals’ house of the University of Veterinary and Animal Sciences, Lahore (UVAAS). Rats were housed in standard polypropylene cages (41 × 34 × 16 cm) at 25 ± 2°C temperature and 65 ± 5% humidity in a 12‐h light/dark cycle with water ad libitum and standard rat chow freely available to the animals. The animals were distributed randomly and a maximum of six rats were kept in each cage.

#### 2.6.2 Acute oral toxicity study

Organization for Economic Co‐operation and Development (OECD) guidelines‐425 were followed for the acute toxicity study of extract (OECD, 2001). The rats were fasted 12 h prior to the experiments with free access to water. The hibiscus and ginger extract were administered at doses of 50, 300, 500, and 2000 mg/kg/p.o., and the behavioral change was observed up to 24 h. Both the extracts were found to be nontoxic up to the maximum dose of 2000 mg/kg body weight. Doses selected for in vivo antioxidant and antiobesity study were 250 and 500 mg/kg, respectively.

#### 2.6.3 **Composition of normal and high‐fat**–**sugar diet formula**

The normal diet (standard rat chow) was purchased locally and contained corn starch (50%), casein (26%), sucrose (9%), cellulose (5%), corn oil (5%), mineral mixture (4%), and vitamin mixture (1%). The high‐fat and high‐sugar diet was prepared in a pellet form and contained beef tallow (40%), casein (26%), corn starch (15%), sucrose (9%), cellulose (5%), mineral mixture (4%), and vitamin mixture (1%). The high‐fat–sugar diet group animals were also administered coke^®^ (soft drink) (containing total sugar 10.8 g/100 ml, carbohydrates 10.5 g/100 ml, and sodium 20 g/100 ml) solution (50:50) in water. Thus, the high‐fat diet was a hyper‐caloric diet as compared with the normal diet and contained more lipids with an energy difference of 4.37 kJ/g. Food was stored in the dark at 24°C to avoid the oxidation of fat.

#### 2.6.4 Experimental design

The rats were acclimatized in the animal transit room for 1 week and were then randomly distributed into the following 11 groups (*n* = 6 each). The control group was provided water ad libitum, while all other groups were provided soft drink:water (1:1) solution ad libitum throughout the study period. The orlistat or water extract solution of selected plants was given to relevant groups through oral gavage.

Normal Control (NC) group [Received normal feed (approx. 20 g/rat/day)]

High‐fat and sugar diet control (HFSDC) group [Received HFD (approx. 20 g/rat/day)]

Positive control (PC) group [Received HFSD (approx. 20 g/rat/day) plus orlistat 250 mg/kg body weight/day for 28 days]

H‐250 group [Received HFSD (approx. 20 g/rat/day) supplemented with hibiscus extract (250 mg/kg BW/day for 28 days)]

H‐500 group [Received HFSD (approx. 20 g/rat/day) supplemented with hibiscus extract (500 mg/kg BW/day for 28 days)]

G‐250 group [Received HFSD (approx. 20 g/rat/day) supplemented with ginger extract (250 mg/kg BW/day for 28 days)]

G‐500 group [Received HFSD (approx. 20 g/rat/day) supplemented with ginger extract (500 mg/kg BW/day for 28 days)]

HG‐250 group [Received HFSD (approx. 20 g/rat/day) supplemented with hibiscus:ginger (3:1) extract (250 mg/kg BW/day for 28 days)]

HG‐500 group [Received HFSD (approx. 20 g/rat/day) supplemented with hibiscus:ginger (3:1) extract (500 mg/kg BW/day for 28 days)]

### 2.7 Observations recorded

#### 2.7.1 Obesity parameters

Body weight gain (%) and body mass index (BMI) were calculated as indicators of obesity. Individual body weight of each rat was recorded at days 0, 7, 14, 21, and 28 and the average weight gain of each group was calculated as

$$\text{Weight}\;\text{increase}\left(\%\right)=\frac{\text{Weight}\;\text{at}\;\text{day}\;28‐\text{Weight}\;\text{at}\;\text{day}\;0\left(g\right)}{\text{Weight}\;\text{at}\;\text{day}\;0\left(g\right)}\times 100$$

BMI was calculated at the end of the experiment by dividing the rat weight in g by the rat length (from nasal to anal region) in cm^2^ (Chien et al., 2016).

#### 2.7.2 Collection of blood sample

At the end of each experiment, the animals were fasted for 12 h but allowed free access to water. Blood samples were taken (4 ml) from the right carotid artery, under chloroform anesthesia, into a tube and centrifuged for 15 min at 3000 rpm. The clear layer of plasma was transferred into microcentrifuge tubes, labeled, and stored at −70°C until biochemical investigation (Ismail, 2014). Upon completion of blood collection, the animals were euthanized by exsanguinations under chloroform anesthesia.

#### 2.7.3 Collection of kidneys and liver

After collecting the blood, kidneys and liver were rapidly dissected and placed in petri dishes containing normal saline. The organs were cleared from connective tissues and blood clots, weighed, and then stored in 10% formalin until histological examination was performed (Chien et al., 2016).

The kidney index (KI) was calculated from body and average kidneys’ weights using the equation

$$\text{KI}\left(\%\right)=\frac{\text{Average}\;\text{kidney}\;\text{weight}\left(g\right)}{\text{Rat}\;\text{weight}\left(g\right)}\times 100$$

Similarly, liver index (LI) was calculated from body and liver weights using the equation

$$\text{LI}\left(\%\right)=\frac{\text{Liver}\;\text{weight}\left(g\right)}{\text{Rat}\;\text{weight}\left(g\right)}\times 100$$

### 2.8 Biochemical investigations

#### 2.8.1 Estimation of cholesterols

Serum was used for the estimation of following biochemical parameters using a semi auto analyzer, as reported by Goyal et al. (2006). Total cholesterol (TC) was estimated by the cholesterol esterase method, triglyceride (TGL) was estimated by the glycerol‐3‐phosphate oxidase method, high‐density lipoprotein (HDL) cholesterol by the phosphotungstate method using respective diagnostic kits obtained from Bayer Diagnostics Ltd, Pakistan. Low‐density lipoprotein (LDL) and very low‐density lipoprotein (VLDL) cholesterols were calculated as per Friedewald's formulae (Goyal et al., 2006), whereas the atherogenic index was calculated by using the method described by Muruganandan et al. (2005):

$$\begin{array}{c} \text{LDL}\;\text{cholesterol}\left(\frac{\text{mg}}{\text{dL}}\right)=\text{Total}\;\text{serum}\;\text{cholesterol}‐\text{HDL}\;\text{cholesterol}\\ \;\;\;\;\;‐\frac{\text{Total}\;\text{serum}\;\text{triglycerides}}{5} \end{array}$$

$$\text{VLDL}\;\text{cholesterol}\left(\frac{\text{mg}}{\text{dL}}\right)=\frac{\text{Total}\;\text{serum}\;\text{triglycerides}}{5}$$

$$\text{Atherogenic}\;\text{index}=\frac{\text{Total}\;\text{serum}\;\text{cholesterol}‐\text{HDL}\;\text{cholesterol}}{\text{HDL}\;\text{cholesterol}}$$

#### 2.8.2 Estimation of liver and kidney functions

Serum creatinine and alkaline phosphate were determined as indicator of kidney function, while serum alanine aminotransferase (ALT), aspartate aminotransferase (AST), and bilirubin total (BT) levels were measured as indicators for liver function. All these biochemical assays were performed on an auto analyzer (Opera, Techicon, Bayer, and USA) (İşeri et al., 2007).

#### 2.8.3 Estimation of oxidative stress parameters

The oxidative status of the animals was determined by conducting a series of tests from the serum samples. Oxidative damage of lipids was assessed by estimating the lipid peroxidation product, malondialdehyde (MDA), as reported by Ohkawa et al. (1979) with minor changes. Both nonenzymatic and enzymatic defense against oxidative stress was studied by measuring reduced glutathione (GSH) and superoxide dismutase (SOD) levels using reported protocols with minor modifications (Kakkar et al., 1984). The sum of endogenous antioxidant activity was determined by estimating the total antioxidant capacity (TAC) using the method of Miller et al. (1993).

### 2.9 Histopathology of liver and kidney tissues

Collected tissues, such as liver and kidney of all the groups, were placed in formalin solution (10%) for 3–5 days. Thereafter, tissues were subjected to overnight washing in running tap water to remove tissues from fixative. To remove water from tissues, dehydration process was carried out in serial dilutions of ethyl alcohol followed by clearing with xylene. The tissues were embedded in paraffin wax to make blocks in a special plastic. Tissues were cut into thin sections (5–15 µm thickness) using a microtome. Mayer's egg albumin was used for mounting the tissue sections on labeled glass slides. Finally, the stained slides were observed using a light microscope for morphological studies.

### 2.10 Statistical analysis

All procedures were performed in three replicates and the results are presented as mean ± standard deviation of three independent experiments. Statistical analysis was performed by means of the statistical package, STATISTICA (Stat Sift Inc.). Data from different tests were analyzed using one‐way and two‐way analysis of variance (ANOVA) followed by the Bonferroni/Dunnett (all mean) post hoc test and the differences between the means were considered statistically significant at *p* ≤ .05 (Hussain et al., 2013). Linear regression analysis and analysis of covariance (ANCOVA) were performed by using SPSS (Version 16).

## 3 RESULTS AND DISCUSSION

### 3.1 Extract yields and in vitro antioxidant potentials

Aqueous extract yields (g/100 g) of *Hibiscus rosa‐sinensis* and *Zingiber officinalis* on dry plant material basis are given in Table 1. The maximum yield of extract (18.41 g/100 g) was found from *Hibiscus rosa‐sinensis* and the minimum from *Zingiber officinalis* (10.22 g/100 g). Aqueous extract yield depends on the amount of extractable polar compounds in plant materials (Sobhy et al., 2017; Sultana et al., 2007).

**TABLE 1:** Extract yield, total phenolic, total flavonoid contents, and radical scavenging capacity of different herbal teas

Assays	Herbal Teas		BHT
	Hibiscus	Ginger	
Extract yield (g/100 g)	18.41 ± 0.55^b^	10.22 ± 0.51^a^	‐‐‐
TPC (mg/g of dry plant material, measured as gallic acid equivalent)	5.82 ± 0.33^b^	1.45 ± 0.08^a^	‐‐‐
TFC (mg/g of dry plant material, measured as catechin equivalent)	9.17 ± 0.90^b^	1.95 ± 0.13^a^	‐‐‐
DPPH radical scavenging activity (%) exhibited by 10 µg/ml	59.0 ± 2.0^b^	53.4 ± 1.4^a^	89.1 ± 2.2^c^

Note

Values are mean ± SD of three independent experiments.

Different superscript letters in the same row represent significant (*p* ≤ .05) difference among hibiscus, ginger teas, and synthetic antioxidant (BHT).

Total phenolic (TP) and total flavonoid (TF) contents of both the herbal extracts are presented in Table 1. The aqueous extract of *Hibiscus rosa‐sinensis* showed a higher concentration of TPC (5.82 mg/g of dry plant material measured as gallic acid equivalent) and TFC (9.17 mg/g of dry plant material, measured as catechin equivalent). *Zingiber officinalis* extract showed 1.45 mg/g and 1.95 mg/g TPC and TFC, respectively. Free radical scavenging activity of various herbal extract solutions (10 µg/ml) was measured by the DPPH radical scavenging assay and the results are presented in Table 1. *Hibiscus rosa‐sinensis* tea exhibited more radical scavenging activity (59.0%) than ginger tea (53.4%). When compared with the synthetic antioxidant butylhydroxytoluene (BHT) (89.1%), herbal teas offered lower radical scavenging capacity. The statistical analysis showed that the difference in TRC, TFC, and DPPH radical scavenging capacity of herbal teas was significant (*p* ≤ .05).

Phenolic compounds are present in almost every plant and have physiological and morphological importance due to their antioxidant potentials (Kanatt et al., 2014). Hence, it is essential to quantify phenolic contents and to assess its contribution against oxidative stress (Oh et al., 2013). Afify and Hassan (2016) evaluated TPC and TFC from aqueous hibiscus flowers extract, which were 2.35 and 0.31 mg/g of flowers, respectively. Tohma et al. (2017) reported 52.8 µg TPC per mg GAE and 3.9 µg TFC per mg quercetin equivalent in water extract of ginger. The variation in the TPC and TFC compared with the findings of the previous study might have been due to the differences in agro‐climatic, geographical, and seasonal conditions. DPPH free radical scavenging capacity increases when the extract concentration increases due to an increase in the concentration of phenolic compounds (Sultana et al., 2007).

### 3.2 HPLC analysis for phenolic acids and flavonoids

**FIGURE 1:**
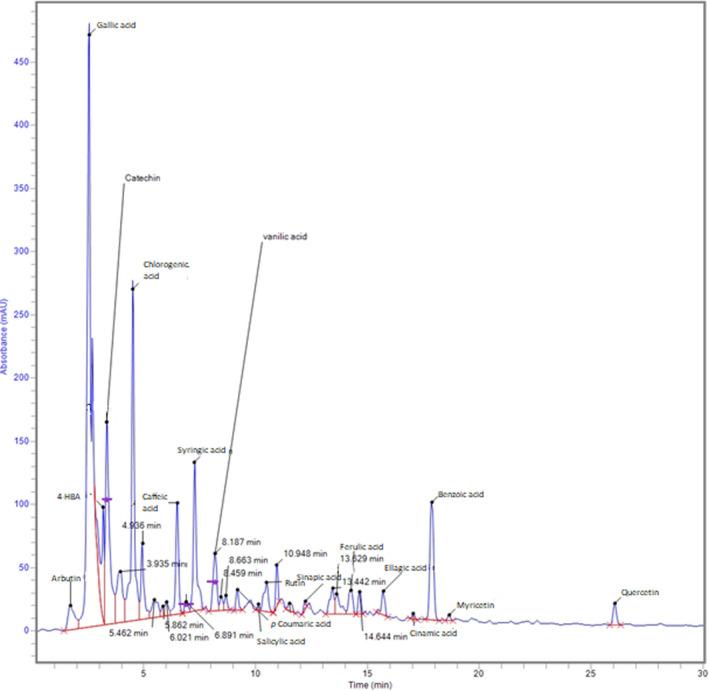
Typical HPLC chromatogram showing the separation of phenolic acids and flavonoids from hibiscus extracts

Phenolic acids and flavonoids were quantified using the RP‐HPLC method. The developed HPLC method could separate 15 phenolic acids and four flavonoids simultaneously within 25 min at a flow rate of 0.8 ml/min (Figure 1). The concentration (mg/100 ml of herbal tea) of 15 phenolic acids, including gallic acid, chlorogenic acid, salicylic acid, caffeic acid, 4‐hydroxy benzoic acid, arbutin, *p*‐coumaric acid, syringic acid, vanillic acid, sinapic acid and ferulic acid, ellagic acid, cinnamic acid, benzoic acid, tannic acid, and three flavonoids, including catechin, myricetin, and quercetin in *Hibiscus rosa‐sinensis* and *Zingiber officinalis* tea is presented in Table 2. Gallic acid (55.21 and 17.32 mg/100 ml) was the major phenolic acid found in hibiscus and ginger teas and catechin (78.25 and 102.8 mg/100 ml) was the major flavonoid followed by rutin (15.34 and 83.37 mg/100 ml). *Hibiscus rosa‐sinensis* extract also contained chlorogenic acid (14.39 mg/100 ml) and caffeic acid (8.43 mg/100 ml), while ginger tea contained 4‐hydroxybenxoic acid (24.18 mg/100 ml), salicylic acid (8.99 mg/100 ml), and arbutin (7.66 mg/100 ml). The variation in the contents of phenolic acids and flavonoids was found to be significant (*p* ≤ .05) between the two teas.

**TABLE 2:** Contents of phenolic acids and flavonoids identified from aqueous herbal extract by Rp‐HPLC

Compounds	Concentration (mg/100 ml of tea)	
	Hibiscus	Ginger
Tannic acid	ND	0.21 ± 0.01
Arbutin	1.39 ± 0.05^a^	7.66 ± 0.40^b^
Gallic acid	55.21 ± 2.70^d^	17.32 ± 0.53^a^
4‐Hydroxybenzoic acid	3.60 ± 0.19^b^	24.18 ± 1.12^d^
Catechin	78.25 ± 3.92^a^	102.8 ± 3.94^b^
Chlorogenic acid	14.39 ± 0.70^b^	8.14 ± 0.42^a^
Caffeic acid	8.43 ± 0.41^d^	0.83 ± 0.05^a^
Syringic acid	1.83 ± 0.04^c^	1.49 ± 0.06^b^
Vanillic acid	1.24 ± 0.07^b^	0.95 ± 0.04^a^
*p*‐Coumaric acid	3.92 ± 0.17^b^	2.63 ± 0.11^a^
Salicylic acid	0.39 ± 0.02^a^	8.99 ± 0.34^d^
Rutin	15.34 ± 1.01^a^	83.37 ± 3.39c
Sinapic acid	0.23 ± 0.02^a^	0.22 ± 0.01^a^
Ferulic acid	1.35 ± 0.05^c^	0.17 ± 0.01^a^
Ellagic acid	0.89 ± 0.06^a^	1.63 ± 0.06^c^
Cinnamic acid	0.19 ± 0.01^a^	0.33 ± 0.02^b^
Benzoic acid	4.72 ± 0.30^c^	ND
Myricetin	0.20 ± 0.01^a^	ND
Quercetin	1.74 ± 0.07^a^	ND

Note

Values are mean ± SD of three independent experiments.

Different superscript letters in the same row represent significant (*p* ≤ .05) difference between hibiscus and ginger teas.

Flowers, due to accumulation of phenolic compounds, possess comparatively higher amounts of flavonoid contents than other plant parts. Purushothaman et al. (2016) reported that the methanol extract of hibiscus flower contained quercetin (7.6 μg/g), kaempferol (361.9 μg/g), and myricetin (50.7 μg/g). Tohma et al. (2017) identified eight different phenolic acids in the water extract of ginger, among which *p*‐hydroxybenzoic acid (321.6 mg/kg), ferulic acid (88.8 mg/kg), and *p*‐coumaric acid (291.4 mg/kg) were more abundant in lyophilized aqueous ginger extract. Our results are also in agreement with findings in the literature and some variation might be due to variation in extraction techniques, drying parameters, geographical, and seasonal conditions of the samples.

### 3.3 **Effect of herbal teas on various parameters of high‐fat**–**sugar diet‐induced obesity model**

#### 3.3.1 Effect on body weight

The initial and final body weight, percent increase in body weight, and BMI of control and treated rat groups are presented in Table 3. The high‐fat and sugar diet control (HFSDC) group showed a significant (*p* ≤ .05) increase in the rats’ body weight and BMI, thus indicating the effectiveness of the high‐fat–sugar diet‐induced obesity model. The HFSDC group presented an increase of 99.30% of body weight when compared with the normal diet control group (40.64%). The BMI of the HFSDC group was 0.79 g/cm^2^, which was significantly higher (*p* ≤ .05) than that of the NC group (0.60 g/cm^2^). The results revealed that the oral administration of all herbal teas and orlistat drug significantly (*p* ≤ .05) decreased the percent increase in body weight and BMI in all the treatment groups. Among all treatment groups, the higher dose of hibiscus (H‐500) showed a less increase in body weight (57.14%) as compared with the HFSDC group and also less BMI (0.50 g/cm^2^), and results are comparable to the positive control group, that is, orlistat group (PC), while the lower dose of ginger (G‐250) showed the least protective effect against obesity (Table 3).

**TABLE 3:** Effect of herbal tea and orlistat on the body, kidney and liver weights, body mass, kidney and liver indices of different groups of obesity rat model

Groups	Body weight			BMI (g/cm^2^)	Kidney weight (g)	Kidney index (%)	Liver weight (g)	Liver index (%)
	Initial (g)	Final (g)	Increase (%)					
NC	155 ± 15	218 ± 12	40.64^#^	0.60 ± 0.05^#^	1.56 ± 0.24	0.71	6.92 ± 1.11	3.17^#^
HFSDC	143 ± 14	285 ± 12	99.30*	0.79 ± 0.05*	1.74 ± 0.17	0.61*	10.1 ± 1.00	3.54*
PC	145 ± 11	228 ± 21	57.24*^#^	0.57 ± 0.05^#^	1.57 ± 0.23	0.68^#^	8.13 ± 1.13	3.45
H‐250	132 ± 14	226 ± 18	71.21*^#^	0.51 ± 0.03*^#^	1.57 ± 0.23	0.64	7.76 ± 1.01	3.43
H‐500	147 ± 13	231 ± 11	57.14*^#^	0.50 ± 0.04*^#^	1.55 ± 0.25	0.67	6.24 ± 1.09	3.41^#^
G‐250	146 ± 10	264 ± 16	80.82*	0.66 ± 0.05^#^	1.71 ± 0.18	0.63	8.69 ± 1.04	3.46*
G‐500	149 ± 13	244 ± 10	63.75*^#^	0.61 ± 0.05^#^	1.64 ± 0.21	0.66	7.94 ± 1.11	3.41^#^
HG‐250	147 ± 12	259 ± 11	76.19*^#^	0.59 ± 0.04^#^	1.59 ± 0.10	0.61	8.93 ± 0.88	3.45*
HG‐500	147 ± 11	232 ± 11	57.82*^#^	0.53 ± 0.01*^#^	1.48 ± 0.20	0.64	7.88 ± 1.00	3.40^#^

Note

Values are mean ± standard deviation of six rats of the same group.

Abbreviations: BMI, body mass index; G‐250 and G‐500, ginger 250 and 500 mg/kg BW; H‐250 and H‐500, hibiscus 250 and 500 mg/kg BW; HFSDC, high‐fat and sugar diet control; HG‐250 and HG‐500, hibiscus:ginger (3:1) 250 and 500 mg/kg BW; NC, normal control.

PC, positive control.

*Significant (*p* ≤ .05) difference compared with NC.

#Significant (*p* ≤ .05) difference compared with HFSDC among all the groups.

A higher dose of mixed tea (hibiscus and ginger 3:1) showed a 57.82% increase in body weight and 0.53 g/cm^2^ BMI, thus showing more protective effect than ginger tea alone. Overall, the higher doses of extracts showed a better antiobesity potential as compared with lower doses in terms of BMI and weight gain.

The cause of obesity is manifold, but the most common cause is still the dietary factor, especially the consumption of high‐fat and sugar diet (Chien et al., 2016). BMI, which is defined as “weight in kg/square of height in meters (kg/m^2^),” is an important parameter to assess the weight gain and obesity (WHO, 2017). A BMI of 30 kg/m^2^ or higher is normally considered obese, while 25 kg/m^2^ or higher BMI is considered overweight (WHO, 2017). Consumption of HFSD increases the fat mass due to the augmentation of triglyceride storage in adipose tissues after fatty acid synthesis in the liver (Hernández‐Saavedra et al., 2016). Increase in the body weight in high‐fat and sugar diet rats group of the present study may be due to the consumption of diet rich in energy in the form of saturated fats and high calorie drinking water which would have resulted in fat deposition in various body fat pads of rats. According to Velez‐Carrasco et al. (2008), 5%–10% reduction in body weight can have a significant effect on the health status. However, the reduced body weight in orlistat‐treated groups is because of a selective reduction in body fat, leaving lean mass unchanged (Mahmoud & Elnour, 2013). The results of the present study are in line with the findings of Nammi et al. (2009) who reported that ethanol extract of *Z. officinalis* (100–400 mg/kg) significantly suppresses body weight gain by 17%–23% after 3–4 weeks compared with the high‐fat diet group. In another study, Nazish et al. (2016) reported that administration of 20 mg/kg aqueous extract of *Z. officinalis* significantly decreases the body weight of rats fed a high‐fat diet. Similarly, the aqueous extract of ginger (200 mg/kg) produced an antiobesity effect in obese diabetic rats (Ismail, 2014). Mahmoud and Elnour (2013) reported that the administration of 5% ginger powder to rats fed a high‐fat diet significantly decreases body weight of rats. The reduction in the rat body weight due to herbal extract treatment may be due to the inhibitory action of ginger on the absorption of dietary fats (Gerald et al., 2008). No study is available in the literature on the antiobesity effect of *Hibiscus rosa sinensis* tea on rat body weight reduction; however, the antiobesity effect of *Hibiscus sabdariffa* aqueous extract was demonstrated by the significant reduction in weight gain between the treated and nontreated rat groups, which was dose dependent (Omar et al., 2018).

#### 3.3.2 Effect on organ weights

Liver and kidney weights of the control, HFSDC, and all the treatment groups were recorded and are presented in Table 3. The HFSDC group showed a significant increase (*p* ≤ .05) in liver and kidney weights as compared with the NC group. The higher doses of all herbal extracts (500 mg/kg) significantly reduced the organ weights and kidney and liver indices, whereas lower (250 mg/kg) doses did not show significant changes in this aspect. To our knowledge, none of the previous studies have reported the effects of *Hibiscus rosa sinensis* tea/extract on the kidney and liver indices. However, Nazish et al. (2016) reported a decrease in the weight of liver and kidney in the *Z. officinalis‐*treated albino rat group as compared with control and high‐fat diet‐fed rat groups.

#### 3.3.3 Effect on serum lipid profile

**FIGURE 2:**
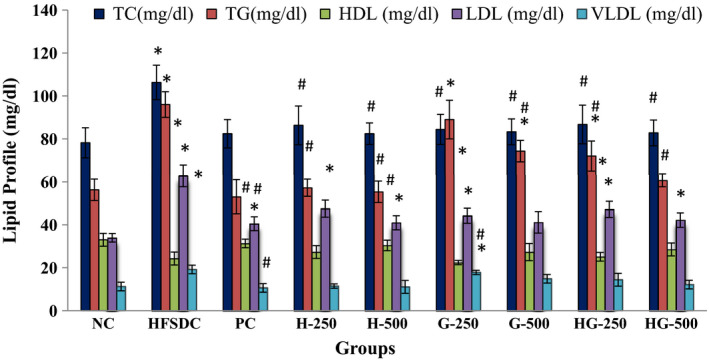
Effect of treatment on the lipid profile of different rat groups. HDL, high‐density lipoprotein; LDL, low‐density lipoprotein; TC, total cholesterol; TG, triglycerides; VLDL, very low‐density lipoprotein. *Significant (*p* ≤ .05) difference compared with NC. #Significant (*p* ≤ .05) difference compared with HFSDC among all the groups

The lipid profile of NC, HFSDC, and all the treatment groups is presented in Figure 2. The HFSDC group had significantly (*p* ≤ .05) increased serum levels of TC (106.3 mg/dl), TG (96.0 mg/dl), LDL (62.8 mg/dl), and VLDL (19.2 mg/dl) and had decreased levels of HDL (24.3 mg/dl), when compared with the NC and treatment groups. All the treatment groups showed the protective effect as measured by levels of serum TC, TG, LDL, VLDL, and HDL. Maximum protection effect was shown by hibiscus extract (500 mg/kg BW) and TC, TG, HDL, LDL, and VLDL levels of H‐500 group were found to be 82.4, 55.4, 30.4, 40.9, and 11.1 mg/dl, respectively, which were comparable to the PC group. Lower dose of ginger extract showed the least protective effect and TC, TG, HDL, LDL, and VLDL levels of G‐250 group were found to be 84.4, 89.0, 22.4, 44.2, and 17.8 mg/dl, respectively. The atherogenic index of HFSDC was significantly (*p* ≤ .05) increased than that of the NC group and all the treatment groups significantly (*p* ≤ .05) reduced the atherogenic index value of all the groups, which showed the protective effect of hibiscus, ginger, and mixed teas at both 250 and 500 mg/kg doses (Table 4). Overall, all the higher doses of extracts showed a better protective effect than the lower doses.

**TABLE 4:** Effect of herbal tea and orlistat treatment on the biochemical parameters of different groups of obesity rat model

Groups	Liver parameters			Kidney parameters		Atherogenic index
	BT (mg/dl)	AST (µ/L)	ALT) (µ/L)	AP (µ/L)	SC (mg/dl)	
NC	0.43 ± 0.05^#^	63.0 ± 5.0^#^	81.2 ± 5.3	143 ± 12^#^	0.43 ± 0.06^#^	1.37 ± 0.08^#^
HFSDC	0.26 ± 0.02*	98.4 ± 4.8*	82.1 ± 4.0	164 ± 9^*^	0.55 ± 0.04*	3.37 ± 0.15*
PC	0.37 ± 0.03^#^	62.5 ± 5.8^#^	62.1 ± 3.9*^#^	152 ± 11	0.41 ± 0.06^#^	1.63 ± 0.10*^#^
H‐250	0.33 ± 0.03*^#^	79.9 ± 3.2*^#^	74.7 ± 4.6	146 ± 15	0.46 ± 0.03^#^	2.16 ± 0.11*^#^
H‐500	0.38 ± 0.03^#^	66.6 ± 2.6^#^	69.0 ± 3.1*^#^	142 ± 11^#^	0.45 ± 0.04^#^	1.71 ± 0.11*^#^
G−250	0.37 ± 0.03^#^	87.3 ± 4.7*^#^	79.0 ± 4.4	147 ± 16	0.43 ± 0.03^#^	2.77 ± 0.18*^#^
G‐500	0.41 ± 0.03^#^	75.5 ± 3.3*^#^	78.6 ± 2.8	146 ± 15	0.38 ± 0.03^#^	2.05 ± 0.19*^#^
HG‐250	0.35 ± 0.03^#^	82.0 ± 4.1*^#^	77.1 ± 4.0	148 ± 11	0.44 ± 0.06^#^	2.44 ± 0.10*^#^
HG‐500	0.38 ± 0.04^#^	70.3 ± 6.0^#^	70.3 ± 2.7*^#^	143 ± 10^#^	0.39 ± 0.03^#^	1.99 ± 0.09*^#^

Note

Values are mean ± standard deviation of six rats of the same group.

Abbreviations: ALT, alanine aminotransferase; AP, alkaline phosphatase; AST, aspartate aminotransferase; BT, bilirubin total; G‐250 and G‐500, ginger 250 and 500 mg/kg BW; H‐250 and H‐500, hibiscus 250 and 500 mg/kg BW; HFSDC, high‐fat and sugar diet control; HG‐250 and HG‐500, hibiscus:ginger (3:1) 250 and 500 mg/kg BW; NC, normal control; PC, positive control; SC, serum creatinine.

*Significant (*p* ≤ .05) difference compared with NC.

#Significant (*p* ≤ .05) difference compared with HFSDC among all the groups.

There are some reports available in the literature on the effect of herbal teas on blood cholesterols levels (El‐Rokh et al., 2010; Gomathi et al., 2008; Saluja et al., 1978). The results of the present study are similar to the findings of El‐Rokh et al. (2010) and Ismail (2014), who found that the aqueous extracts of ginger lowered serum total cholesterols, triglycerides, and low‐density lipoprotein. The TG‐lowering effect of ginger may be due to its ability to enhance lipase activity (El‐Rokh et al., 2010). Gomathi et al. (2008) and Sikarwar and Patil (2011) reported that the flowers of hibiscus significantly reduced the LDL and VLDL cholesterols and increased the HDL cholesterol level. *Hibiscus rosa‐sinensis* reduced the LDL cholesterol of monosodium glutamate‐induced obesity group from 46.75 to 22.92 mg/dl (Gomathi et al., 2008). Investigated teas are rich in flavonoids and phenolic acids and it has been reported that the hypolipidemic effect of various herbal extracts may be related to the ability of various phenolic acids and flavonoids by stimulation of thermogenesis and decreased fat accumulation, which can be related to their pancreatic lipase inhibitory activity (Hernández‐Saavedra et al., 2016). Moreover, flavonoids also inhibit the absorption of dietary cholesterol and decrease in serum cholesterol (Saluja et al., 1978). High levels of LDL and TC increase the risk of coronary heart diseases, while an increase in HDL cholesterol is supportive in transporting overloaded cholesterol to the liver for excretion in the bile (Saravanan et al., 2014). The increase in LDL cholesterol may be due to the reduced expression or activity of the LDL‐receptor sites in response to a high‐fat diet treatment (Nammi et al., 2009). The increase in LDL‐cholesterol may be due to the reduced expression or activity of the LDL‐receptor sites in response to treatment groups (Nammi et al., 2009). Therefore, lowering the LDL‐cholesterol level may be an important factor in lowering the serum total cholesterol level in rats fed a high‐fat diet. The reduction of LDL‐cholesterol by herbal teas treatments could be due to prevention of the suppressive action of high‐fat diet on the LDL‐receptor site. The change in lipid profile levels induced by a high‐fat diet might be due to the activation of gastric lipases and enhanced intestinal fat absorption, while an increase in triglyceride levels was due to the dietary cholesterol that reduced fatty acid oxidation which, in turn, increased the levels of hepatic triglycerols (Saravanan et al., 2014). The more the atherogenic index, the higher is the risk of oxidative damage of kidneys, liver, heart, aorta, and coronaries due to fatty infiltration, plaque, foam cells, and/or lipids (Mehta et al., 2003).

#### 3.3.4 Effect on plasma levels of liver and kidney enzymes

Liver parameters like bilirubin total (BT), alanine aminotransferase (ALT), aspartate aminotransferase (AST) and kidney parameters like serum creatinine (SC) and alkaline phosphatase (AP) of all the control and treatment groups were studied and are presented in Table 4. Rats fed on HFSD for 4 weeks had significantly increased serum levels of AST and decreased total bilirubin level, when compared with the normal control group, thus showing the effectiveness of the model. A decline in the levels of ALT and AST and an increase in the BT were recorded in all the treatment groups. The major effect appeared in the H‐500 group when compared with the PC group. Alkaline phosphate (AP) and serum creatinine (SC) significantly (*p* ≤ .05) increased in the HFSDC group in comparison with the control group. All the extracts showed a protective effect and the increased levels of the AP and SC decreased in all treated groups. The maximum protective effect was recorded in H‐500 groups which were comparable to the PC group (Table 4). Ginger extract showed a better effect in the SC level as compared with others.

In obesity, generally, oxidative stress is increased causing an increase in the bilirubin consumption leading to reduced serum bilirubin level (Karadag et al., 2017). Ismail (2014) also reported that the ginger water extract significantly decreased the elevated serum levels of liver enzymes (ALT, AST) in obese rats. Sanadheera et al. (2021) reported decreased ALT level in pregnant diabetic Wistar rats fed *Hibiscus rosa‐sinensis* aqueous extract at a dose of 100–400 mg/kg. In another study, Jenko‐Pražnikar et al. (2013) reported that the serum bilirubin levels were negatively associated with abdominal obesity. Chang et al. (2012) reported that serum bilirubin levels were inversely associated with LDL, TC, and TG and positively associated with HDL.

#### 3.3.5 Effect on oxidative stress parameters

The effects of hibiscus, ginger, and mixed extracts on the levels of malondialdehyde (MDA), superoxide dismutase (SOD), reduced glutathione (GSH), and total antioxidant capacity (TAC) of all the treatments and control groups were assessed and are presented in Table 5. The level of MDA in the serum of rats of high‐fat and sugar diet control (HFSDC) group was 6.97 nmol/L, which is significantly (*p* ≤ .05) higher than that of the NC group (2.73 nmol/L), thus showing the oxidative stress in tested animals. All the treatment groups, including the PC group, showed a significant (*p* ≤ .05) decrease in the alleviated levels of MDA. Daily administration of hibiscus, ginger, and mixed teas effectively prevented the generation of MDA in rat groups and the best effect (3.17 nmol/L) was shown in hibiscus: ginger‐500 group (500 mg/kg BW/d) followed by hibiscus‐500 group (3.19 nmol/L) and ginger‐500 group (3.29 nmol/L). The protective effect is even better than that of the PC group (3.21 nmol/L). The serum SOD level in the HFSDC group was found to be 120.4 U/ml, which is significantly (*p* ≤ .05) lesser than that of the NC group (158.9 U/ml). Maximum protective effect was observed in the HG‐500 group followed by H‐500 group and the levels of SOD were found to be 142.2 and 135.2 U/ml, respectively. A significant (*p* ≤ .05) reduction in the level of GSH was recorded in the HFSDC group (123.2 mg/L) as compared with the NC group (160.3 mg/L), which leads to oxidative stress. All the treatment groups showed the protective effect against oxidative stress, and the level of GSH in H‐500 group was 167.6 mg/L followed by the HG‐500 group (161.4 mg/L), H‐250 group (161.1 mg/L), and the G‐500 group (158.9 mg/L). The total antioxidant capacity was significantly (*p* ≤ .05) decreased in the HFSDC group (1.36 mmol/L) as compared with the NC group (1.93 mmol/L). The TAC of different treatment groups is mentioned in Table 5. All the treatment groups, except G‐250, significantly (*p* ≤ .05) increased the TAC values. The best protective effect was shown by mixed tea groups (HG‐500 and HG‐250) and the value of TAC was 1.85 and 1.71 mmol/L, respectively. The H‐500 group also showed an increase in the TAC value (1.83 mmol/L).

**TABLE 5:** Effect of herbal tea and orlistat treatment on the oxidative stress parameters of different groups of obesity rat model

Groups	MDA (nmol/L)	SOD (U/ml)	GSH (mg/L)	TAC (mmol/L)
NC	2.73 ± 0.20^#^	158.9 ± 8.1^#^	160.3 ± 11.0^#^	1.93 ± 0.20^#^
HFSDC	6.97 ± 0.41*	120.4 ± 9.0*	123.2 ± 10.0*	1.36 ± 0.09*
PC	3.21 ± 0.18*^#^	137.1 ± 7.7*	148.9 ± 9.31^#^	1.71 ± 0.19^#^
H‐250	3.34 ± 0.32*^#^	135.0 ± 9.4*	161.1 ± 10.8^#^	1.68 ± 0.09^#^
H‐500	3.19 ± 0.19*^#^	139.3 ± 8.3*^#^	167.6 ± 10.0^#^	1.83 ± 0.16^#^
G‐250	3.41 ± 0.24*^#^	129.1 ± 9.5*	146.3 ± 9.01^#^	1.49 ± 0.08*
G−500	3.29 ± 0.38*^#^	135.2 ± 9.0*	158.9 ± 13.7^#^	1.68 ± 0.18^#^
HG‐250	3.32 ± 0.21*^#^	136.7 ± 9.6*	145.8 ± 9.13^#^	1.71 ± 0.20^#^
HG‐500	3.17 ± 0.27*^#^	142.2 ± 8.2^#^	161.4 ± 10.7^#^	1.87 ± 0.19^#^

Abbreviations: G‐250 and G‐500, ginger 250 and 500 mg/kg BW; GSH, reduced glutathione; H‐250 and H‐500, hibiscus 250 and 500 mg/kg BW; HFSDC, high‐fat and sugar diet control; HG‐250 and HG‐500, hibiscus:ginger (3:1) 250 and 500 mg/kg BW; MDA, malondialdehyde; NC, normal control; PC, positive control; SOD, superoxide dismutase; TAC, total antioxidant capacity.

*Significant (*p* ≤ .05) difference compared with NC.

#Significant (*p* ≤ .05) difference compared with HFSDC among all the groups.

Lipid peroxidation is a key biomarker of evaluation of oxidative stress and the determination of MDA reflects the degree of lipid peroxidation, and that indirectly reflects cellular damage (Hosen et al., 2015). The alleviation in the level of MDA in the HFSDC group may be due to the lipid peroxidation that leads to oxidative stress and the decrease in the MDA level in the treatment groups may be due to the antioxidant potential of hibiscus and ginger teas that retard lipid peroxidation. SOD is metalloenzyme that catalyzes the dismutation of superoxide radicals and decreased levels of SOD show the sign of oxidative stress (Wang et al., 2016). GSH is an important antioxidant present in the body and plays a role in tissue injury and damage due to free radicals and peroxides (Hosen et al., 2015). SOD and GSH levels were decreased significantly in the HFSDC group as compared with the normal control group. This drop in SOD and GSH levels indicates oxidative stress in the rats due to destruction of H_2_O_2_ clearance and validates the notion of hydroxyl radical (•OH) formation (Hosen et al., 2015; Wang et al., 2016). All the treatment groups including the herbal extract groups restored the reduced levels of SOD and GSH, thus showing the potential against oxidative stress and refurbishment of lipid peroxidation. Previous reports also presented that oxidative stress distorted the activity of the endogenous enzymes which play a major role in scavenging toxic free radicals (Bakır et al., 2016; Hosen et al., 2015). TAC is the total effect of various antioxidant compounds and their systemic interactions. Clinically, TAC has been widely used to assess oxidative stress and serum antioxidant depletion (Rice‐Evans & Miller, 1997). Decreased TAC in the HFSDC group rats may be because of either increased oxidative stress or decreased availability of antioxidants. This imbalance was restored with the administration of hibiscus, ginger, and mixed teas, through decreasing free radical generation and increasing antioxidant levels.

### 3.4 Histopathological evidence

**FIGURE 3:**
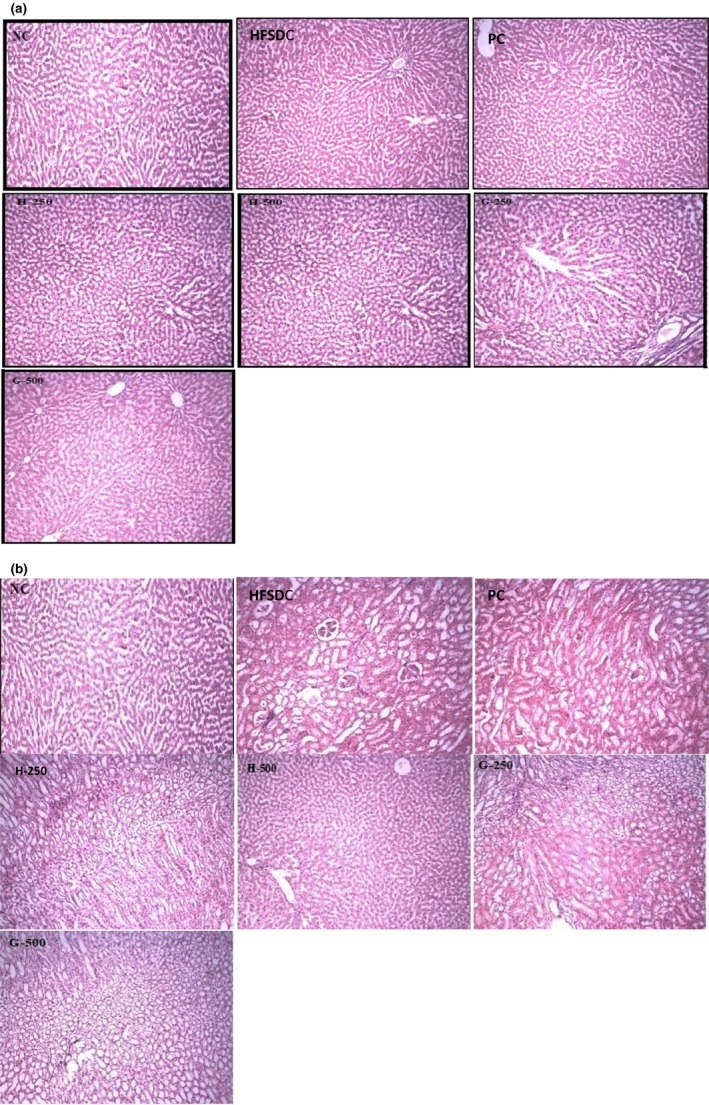
Histopathological microscopic image showing the morphological changes of (a) liver and (b) kidney of different rat groups. G‐250 and G‐500, ginger 250 and 500 mg/kg BW; H‐250 and H‐500, hibiscus 250 and 500 mg/kg BW; HFSDC, high‐fat diet control; NC, normal control; PC, positive control

An acute study showed that all the animals were healthy, agile, with no redness of the eyes, no vocalization, no signs of loss of hair, no moribund and hunchbacked signs after oral administration of extracts. Microscopic examination of the liver and kidney tissues of rats of all groups showed regular architectures with unnoticeable differences in the histological and cellular structures of all the organs except rats of the HFSDC group (Figure 3). Livers of all the treatment groups showed no ballooning, nuclei were of normal shape, and no inflammatory cells were present. Similarly, kidneys of all the groups showed normal glomerulus, tubules, and parenchyma. Mild ballooning and fat droplets were observed in ballooned hepatocytes in the HFSDC group. Histopathological evaluation of biopsy specimens remains the authentic and reproducible diagnostics tool for the fatty liver or other organs (Takahashi & Fukusato, 2014). Hepatocellular ballooning is a sign of hepatocellular injury and is illustrated as swollen hepatocytes with rarefied cytoplasm (Takahashi & Fukusato, 2014).

## 4 CONCLUSION

In conclusion, the use of hibiscus and ginger teas individually and in mixture form at doses of 250 and 500 mg/kg body weight showed a marked reduction in weight gain and oxidative stress in rats fed a high‐fat and high‐sugar diet. The 500 mg/kg dose showed more pronounced effects, which were comparable to the antiobesity drug (orlistat). The investigated teas contained a rich source of phenolic acids and flavonoids, especially gallic acid and catechin. Based on these results, it is concluded that selected herbal infusions may have a therapeutic potential and can be used as antiobesity agents. Further studies should investigate the effects of individual flavonoids and phenolic acids on the biochemical parameters in obesity and elucidate the mechanism of action at the molecular level.

## CONFLICT OF INTEREST

The authors declare that they do not have any conflict of interest.

## ETHICAL APPROVAL

All animal‐related experiments were performed with the prior approval of the Institutional Review Board for Animal Studies (Study No. 19680/IRB No 680), Government College University Faisalabad, Pakistan.

## Data Availability

The data that support the findings of this study are available from the corresponding author upon reasonable request.
